# Giant Cell Tumor of Hoffa’s Fat Pad: A Case Report

**DOI:** 10.31729/jnma.8888

**Published:** 2025-02-28

**Authors:** Rupesh Kumar Vaidya, Prabal Pradhan, Karishma Malla Vaidya

**Affiliations:** 1 Department of Orthopedics, Suvekchya International Hospital, Sitapaila, Kathmandu, Nepal; 2 Department of Pathology, Paropakar Maternity & Women's Hospital, Thapathali, Kathmandu, Nepal

**Keywords:** *benign*, *hoffa's fat pad*, *tenosynovial giant cell tumor*

## Abstract

Tenosynovial giant cell tumor (GCT) is a rare, benign disorder involving the joint's synovial lining, tendon sheath or bursa. It can be classified as localized or diffuse based on its pattern and behavior. Localized form is extremely rare in knee joint. We present an unusual case of localized form of GCT in Hoffa's fat pad in a young female, treated with arthroscopic resection and monitored for over two years with no recurrence. Despite its rarity, GCT of Hoffa's fat pad should be considered in cases of non-traumatic knee pain.

## INTRODUCTION

Tenosynovial giant cell tumor is a rare, benign proliferative disorder involving the joint's synovial lining, tendon sheath or bursa.^1^ It commonly affects young adults and is more prevalent in females.^1^ Byers et al.(1968) classified it into localised and diffuse forms based upon its pattern and behavior. Localized form, also known as GCT of tendon sheath (GCT-TS), typically presents as a single nodule within the tendon sheath or adjacent to joint, while diffuse type (Dt-GCT) demonstrates extensive involvement of synovial lining, bursa and capsule. GCT-TS is common in hand or foot, whereas Dt-GCT tends to primarily involve the lower extremities. GCT-TS is extremely rare in the knee joint.^2^ We present an unusual case of GCT-TS in Hoffa's fat pad in a young female, which was arthroscopically resected and followed up for more than two years with no recurrence.

## CASE

A thirty-eight years old female presented with right knee pain for a year, gradually increasing in intensity and aggravated by movement of the knee. She also noticed a fullness over the patellar tendon which was not increasing in size but painful on touch. She had difficulty in walking rough terrains and sitting crossed leg. She did not remember any trauma on her knees and was on occasional pain killers. Clinical examination revealed mild fullness and tenderness around patellar tendon especially during full flexion. No obvious effusion was present, and stability tests were within normal limits. X rays of the knee were normal.

**Figure 1:**
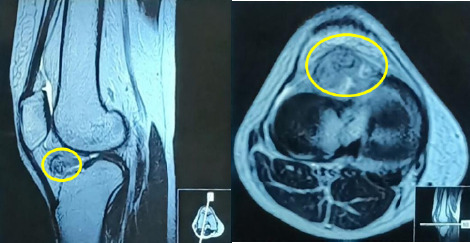
MRI of right knee showing ill-defined soft tissue lesion (marked in yellow) of size 3cm×2.8cm in the anterior joint space

MRI of her right knee showed a 3cm×2.8cm ill-defined soft tissue lesion in the anterior joint space, abutting Hoffa's fat pad, with low T2 signal intensity, suggesting GCT (Figure 1).

**Figure 2:**
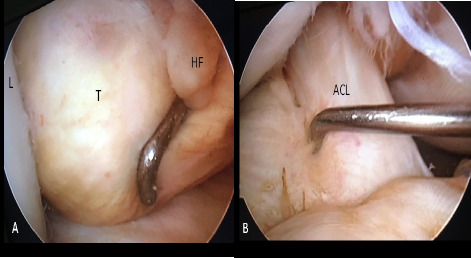
Per-op pictures : Arthroscopic view of the tumor(A) and after excision (B) L:Lateral Condyle, T:Tumor, HF:Hoffa’s Fat Pad, ACL:Anterior Cruciate Ligament

**Figure 3:**
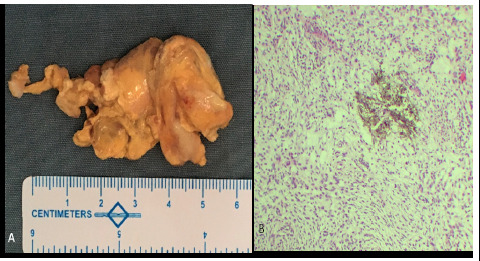
A. Resected mass B. Osteoclast-type giant cells with few hemosiderin-laden macrophages

Due to patient's unrelieved symptoms, arthroscopic resection of the mass was planned. Using standard anterolateral and anteromedial portals, the joint space was inspected in a routine manner. The mass was observed and probed, which was round, soft in texture, around 3 × 3 cm, just posterior to patellar tendon attached to the Hoffa's fat pad with a fibro fatty stalk, encroaching the intercondylar notch, abutting anterior cruciate ligament (ACL) but with no attachments . The mass was excised at the base of the stalk attached to Hoffa's fat pad and removed through anterolateral portal which was enlarged a bit. Any remnant was searched for and the attachment to the fat pad cauterized (Figure 2 and 3).

Histopathological examination revealed round to oval mononuclear cells with mild atypia and some eosinophilic cytoplasm, arranged in a lobular pattern and diffuse sheath, osteoclast-type giant cells, and few hemosiderin-laden macrophages(fig 3B), thus confirming GCT.

Range of motion(ROM) exercise was started on 2nd day post-surgery and gradual partial to full weight bear was allowed within 2 weeks. Suture removal was done at 2 weeks. Patient was followed up at 1, 3, 6, 16 and 28 months. Patient was pain-free at 1 month follow-up. At 16 months, she was asymptomatic and had no complaints, she was performing her full activities without problem. A repeat MRI confirmed no recurrence. At 28 months follow up, she had no further complaints on her knee.

## DISCUSSION

Tenosynovial giant cell tumor is a fibrocystic lesion arising from the synovia of the fibrous tissue surrounding the joints and tendon sheaths. It typically occurs in individuals aged 30-50, with a 2:1 female predominance. Symptoms include a painless or mildly painful mass, limited joint mobility and possible nerve or vessel compression.^3^ There are localized and diffused forms. Localized forms (GCT-TS), usually found in tendons of hands and feet, is extra-articular, while diffused forms (Dt-GCT) involve large synovial joints like knee, ankle and shoulder and are intra-articular.^4^ GCT-TS in patellar tendon or Hoffa's fat pad is very rare. A comprehensive search of literature was carried out for occurrence of GCT-TS in Hoffa's fat pad and very few cases were found. There were 6 cases reported previously from 1994 to 2022.^3,4,5,6,7^ Reported symptoms include joint effusion, painful or painless mass, swelling, tenderness, locking, limitation of knee motion, which can be intermittent or persistent.^1^

Diagnosis of GCT involves clinical evaluation, imaging studies (X-rays, USG or MRI), and biopsy confirmation. X-rays may show soft tissue swelling but often do not directly reveal the tumor. MRI is more effective, showing a well-defined, lobulated mass with intermediate to slightly hyperintense signal on T1-weighted images and variable signal intensity on T2-weighted images due to hemosiderin deposits.^8^

Differential diagnoses of a solitary mass in Hoffa's fat pad include Hoffa's disease, chondroma, osteochondroma, gout, lipoma, fibroma, focal arthrofibrosis, synovial sarcoma, hemangioma, ganglion, and bizarre proliferative osteochondromatous proliferation. These conditions differ from GCT-TS in characteristics such as signal intensity on MRI and the presence of hemosiderin deposits.^8^

Definitive diagnosis of GCT of Hoffa's fat pad relies on histopathological examination of the synovial tissue obtained through surgical resection. Key histological features include polymorphous population of multinucleated osteoclast-like giant cells, epithelioid histiocytes, mononuclear stromal cells and hemosiderin-laden macrophages.^9^

The primary treatment for GCT of Hoffa's fat pad is surgical excision, either arthroscopic or open removal with complete or partial excision of the affected fat pad. Recurrence rate varies from 10-20%.^9^ For aggressive tumors or high recurrence risk, adjuvant therapies like radiotherapy or targeted pharmacotherapy may be considered. Arthroscopy is preferred for localized GCT due to its minimally invasive nature and quicker recovery.^7^ In our case, we preferred arthroscopic excision through standard portals with slight extension of the anterolateral portal as it was localized and in-toto excision was amenable without an open procedure.

There is no data to indicate the frequency and length of follow ups after complete excision of localized GCT of knee. It may vary with the location, initial presentation, growth pattern and persisting symptoms.^10^ In our case, the patient was symptom free after nearly three years of surgery.

## CONCLUSIONS

Although GCT of Hoffa's fat pad is a rare entity, it should be considered as potential cause of non-traumatic knee pain. There are various diagnostic modalities but diagnosis can be confirmed only by histopathological examination. Arthroscopic resection or open excision are the two methods of treatment with possibility of adjuvant therapy in more progressive cases. Surgical technique to be employed, depends on the location, extent and size of the lesion. Recurrence is rare unless the lesion is not excised completely.
